# 

**Published:** 1877-08-20

**Authors:** 


☞All communications relating to the business of THE RECORD, for the year 1877, must be addressed to

DR. R. C. WORD,
Business Manger Southern Med. Rec.,
Atlanta, Ga.

☞Brief and practical communications are solicited on all subjects pertaining to medicine, also reports of cases in practice.

☞Send money by check, postal order or registered letter.

☞Write your name, post-office, county and State plainly.

### COLLABORATORS AND CONTRIBUTORS.

L. B. Anderson, M.D., Va.
Swan M. Burnett, M.D., D. C
J. M. Bristow M.D., Texas
J. W. Booth, M.D., N. C.
W. F. Barr, M.D., Va.
B. W. Corn, M.D., Texas
John Castor, M.D., Iowa.
E. T. Easley. A.M., M.D., Ark
A. H. Goelet, M D., N. Y.
E. C. Gehrung, M.D., Mo.
G. D. Hodge, M.D., Ark.
S. M. Hogan, M.D., Ala.
A. McLane Hamilton, M.D.,NY
C. C. Vanderbeck, M.D., Pa.
Z. B. Herndon, M.D., Va.
Lindsav Johnson, M.D., Ga.
A. R. Kilpatrick, M.D., Tex.
R. Kells, M.D., Miss.
W. L. Lipscomb, M.D., Miss.
J. M. Lewis, M.D., Miss.
G. A. Dyer, M.D., Ind.
C H Lathrop, M.D., I[illegible]
A A Lyon, M.D., La .
M Lewis, M.D., Tenn
G M Rivers, M.D., S C
A S ayne, M.D., Va.
A. M. Whitehead, M. D., O[illegible]

